# Beyond the Diagnosis—Personalized Risk Stratification in Contemporary Cardiovascular Medicine

**DOI:** 10.3390/life16060965

**Published:** 2026-06-08

**Authors:** Jacek Bil

**Affiliations:** National Medical Institute of the Ministry of Interior and Administration, 02-507 Warsaw, Poland; jacek.bil@pimmswia.gov.pl

Cardiovascular disease (CVD) remains the leading cause of mortality worldwide, accounting for approximately 18–20 million deaths annually, with coronary artery disease alone responsible for nearly half of these events [1]. Despite substantial advances in pharmacotherapy, revascularization techniques, and imaging modalities, residual risk remains high: contemporary registries report 1-year mortality rates of 5–10% following acute coronary syndromes (ACS), rising to >20% at 5 years in high-risk populations. These observations underscore a critical limitation of traditional cardiovascular medicine—namely the reliance on diagnosis-centered frameworks that insufficiently capture the heterogeneity of patient risk [2,3].

Cardiovascular medicine is therefore undergoing a paradigm shift—from a discipline focused primarily on disease classification and procedural success toward a more nuanced model centered on individualized risk stratification, multimorbidity assessment, and precision-based clinical decision-making [4]. While traditional diagnostic categories such as ACS, chronic total occlusions (CTOs), peripheral artery disease (PAD), structural heart disease, and cardiac amyloidosis remain fundamental, growing evidence demonstrates that long-term outcomes are more strongly determined by the interplay between procedural complexity, systemic vulnerability, comorbidity burden, and patient-specific biological factors than by the index diagnosis alone [5].

This Special Issue of Life, dedicated to contemporary challenges in cardiovascular medicine, brings together original investigations, reviews, and illustrative case reports that collectively reflect this important paradigm shift. Across diverse cardiovascular settings—from coronary and peripheral interventions to aortic surgery, infiltrative cardiomyopathies, and rare congenital coronary anomalies—the included studies consistently emphasize the importance of moving beyond diagnosis-centered thinking toward integrated and personalized prognostic assessment.

One of the central themes emerging from this issue is the critical importance of procedural complexity and lesion characteristics in determining outcomes after interventional treatment. Karaca et al. explored the prognostic significance of combining anatomical CTO complexity scores with nutritional status in patients undergoing chronic total occlusion percutaneous coronary intervention (CTO-PCI) [6]. In their cohort of 118 patients, adverse outcomes—including mortality and repeat revascularization—were independently associated not only with higher J-CTO scores but also with lower Prognostic Nutritional Index (PNI) values. Importantly, left ventricular ejection fraction, J-CTO score, and PNI emerged as independent predictors of mortality, suggesting that lesion complexity alone is insufficient to fully capture procedural risk. The integration of nutritional and immunological status into preprocedural assessment represents a practical and clinically meaningful extension of conventional CTO risk models. These findings are particularly relevant given that CTO lesions are encountered in approximately 15–20% of patients undergoing coronary angiography, yet procedural success and long-term outcomes remain highly variable across patient subgroups [7]. The incorporation of systemic factors such as nutritional and inflammatory status may therefore improve both patient selection and prognostic precision beyond purely anatomical scoring systems.

A similar message is evident in the field of peripheral endovascular intervention. Unterseeh et al. analyzed determinants of periprocedural mechanical complications during peripheral revascularization in a real-world single-center registry of 283 procedures [8]. Mechanical complications were relatively uncommon (3.2%), with arterial dissection being the most frequent event; however, chronic total occlusions, moderate-to-severe calcification, larger introducer sheath size, and repeated vascular access attempts significantly increased risk. Notably, access route and device type were less influential than lesion complexity and procedural strategy. These findings reinforce the principle that careful lesion preparation and individualized procedural planning remain fundamental to procedural safety, often more so than the choice of device itself. Given that real-world complication rates in peripheral interventions typically range between 2 and 5%, the observed 3.2% incidence aligns with contemporary practice but highlights the disproportionate impact of lesion complexity and operator-dependent factors. This reinforces the concept that procedural risk is not device-driven but context-dependent [9].

The concept of dynamic intraoperative risk assessment is further illustrated by Heuer et al., who investigated the role of near-infrared spectroscopy (NIRS) patterns as predictors of perioperative stroke in patients undergoing surgery for acute type A aortic dissection (ATAAD) [10]. In this cohort of 175 patients, those who developed perioperative stroke demonstrated significantly lower minimum cerebral oxygen saturation during deep hypothermic circulatory arrest, greater NIRS signal variability, and more frequent cerebral desaturation below critical thresholds. These findings support the growing role of functional monitoring—not merely anatomical assessment—in identifying patients at heightened neurological risk. Real-time cerebral oximetry may therefore serve not only as a monitoring tool but also as a prognostic instrument guiding intraoperative decision-making. Perioperative stroke remains a devastating complication of ATAAD surgery, with reported incidence ranging from 5% to 15%, depending on patient profile and surgical strategy. In this context, the identification of intraoperative physiological markers such as NIRS-derived cerebral oxygenation provides an important step toward real-time, modifiable risk stratification [11].

Beyond procedural settings, this Special Issue also highlights the expanding role of advanced imaging-derived markers in chronic cardiovascular diseases. Tsiamis et al. provide a comprehensive review of right ventricular pulmonary artery (RV–PA) coupling in cardiac amyloidosis, emphasizing the prognostic value of the TAPSE/PASP ratio as a simple yet powerful marker of adverse outcomes [12]. While left ventricular function has traditionally dominated prognostic assessment in cardiac amyloidosis, growing evidence suggests that right ventricular dysfunction and ventriculo-arterial uncoupling may better reflect disease progression and heart failure burden. Impaired RV–PA coupling consistently predicted mortality and heart failure hospitalization across both AL and ATTR amyloidosis subtypes. This work reflects a broader trend in cardiovascular imaging: the transition from isolated structural parameters toward integrated physiological assessment that better captures disease severity and clinical vulnerability.

The importance of maintaining broad diagnostic vigilance is elegantly illustrated by the rare case report of adult anomalous left coronary artery from the pulmonary artery (ALCAPA) presented by Veljković et al. [13]. In a young adult with prior myocardial infarction, heart failure symptoms, and reduced left ventricular function, the diagnosis of this rare congenital coronary anomaly required a high index of suspicion and multimodality imaging. Successful surgical correction restored coronary anatomy, although persistent ventricular dysfunction necessitated continued follow-up. Such cases remind clinicians that uncommon etiologies may underlie common presentations and that individualized diagnostic pathways remain essential even in routine cardiology practice.

Finally, the issue directly addresses one of the most important conceptual shifts in contemporary intensive cardiovascular care: the movement from diagnosis-driven prognosis toward vulnerability-based stratification. In our own study evaluating long-term mortality among patients admitted to the cardiac intensive care unit (CICU), we demonstrated that while ACS subtype influenced crude mortality rates, long-term survival was ultimately determined predominantly by age, atrial fibrillation, prior stroke, and overall comorbidity burden rather than by the specific ACS diagnosis itself [14]. Among 825 patients with ACS and complete 5-year follow-up, long-term mortality exceeded 20%, reflecting the high-risk nature of the CICU population. While NSTEMI was associated with the highest unadjusted mortality, multivariable analysis demonstrated that age, atrial fibrillation, prior stroke, and overall comorbidity burden were the dominant predictors of outcome, effectively attenuating the prognostic relevance of ACS subtype.

Taken together, the studies included in this Special Issue reflect a unifying scientific message: precision cardiovascular medicine requires the integration of anatomical complexity, procedural factors, biological vulnerability, imaging-derived functional markers, and comorbidity burden into everyday clinical decision-making. Whether they are treating CTO lesions, managing peripheral interventions, monitoring aortic surgery, assessing infiltrative cardiomyopathies, or stratifying critically ill ACS patients, clinicians are increasingly required to think beyond traditional disease labels.

This conceptual shift has important implications for both clinical research and healthcare systems. Future risk prediction models should move beyond disease-specific variables and adopt multidimensional frameworks integrating clinical, procedural, imaging, and biological data. The incorporation of frailty indices, systemic inflammatory markers, and increasingly omics-based and machine learning-derived predictors may further refine individualized risk assessment. At the same time, large-scale real-world registries and pragmatic studies will remain essential to complement randomized trial data, particularly in capturing multimorbidity and treatment heterogeneity that are often underrepresented in controlled settings [15,16].

In conclusion, the studies presented in this Special Issue collectively illustrate that the future of cardiovascular medicine lies not only in improving procedural success rates, but in refining the precision with which risk is assessed and managed across the entire patient journey. Increasingly, prognosis is shaped less by the diagnostic label itself and more by the interaction between anatomical complexity, systemic vulnerability, and functional reserve. This evolving paradigm aligns with broader trends in medicine toward personalization, data integration, and risk-based decision-making.

Importantly, similar shifts have been observed across major contemporary cardiovascular studies and guidelines, which increasingly emphasize multimorbidity, frailty, and patient-centered outcomes as key determinants of prognosis. Moving beyond diagnosis toward integrated, individualized risk stratification is therefore not merely an incremental improvement—it represents a fundamental transformation in how cardiovascular disease is understood, studied, and treated in modern clinical practice.

## Abbreviations

The following abbreviations are used in this manuscript:

ACS	Acute Coronary Syndromes
AL	Light-chain amyloidosis
ALCAPA	Anomalous Left Coronary Artery from the Pulmonary Artery
ATAAD	Acute Type A Aortic Dissection
ATTR	Transthyretin amyloidosis
CICU	Cardiac Intensive Care Unit
CTO	Chronic Total Occlusion
CTO-PCI	Chronic Total Occlusion Percutaneous Coronary Intervention
CVD	Cardiovascular Disease
J-CTO	Japanese Chronic Total Occlusion score
NIRS	Near-Infrared Spectroscopy
NSTEMI	Non-ST-Elevation Myocardial Infarction
PAD	Peripheral Artery Disease
PCI	Percutaneous Coronary Intervention
PNI	Prognostic Nutritional Index
RV	Right Ventricular
PA	Pulmonary Artery
TAPSE	Tricuspid Annular Plane Systolic Excursion
PASP	Pulmonary Artery Systolic Pressure
